# Ataxia-Telangiectasia Mutated Modulation of Carbon Metabolism in Cancer

**DOI:** 10.3389/fonc.2017.00291

**Published:** 2017-11-29

**Authors:** Erika S. Dahl, Katherine M. Aird

**Affiliations:** ^1^Department of Cellular and Molecular Physiology, Penn State College of Medicine, Hershey, PA, United States

**Keywords:** ataxia-telangiectasia mutated, cellular metabolism, cancer, reactive oxygen species, senescence, p53, AKT, c-myc

## Abstract

The ataxia-telangiectasia mutated (ATM) protein kinase has been extensively studied for its role in the DNA damage response and its association with the disease ataxia telangiectasia. There is increasing evidence that ATM also plays an important role in other cellular processes, including carbon metabolism. Carbon metabolism is highly dysregulated in cancer due to the increased need for cellular biomass. A number of recent studies report a non-canonical role for ATM in the regulation of carbon metabolism. This review highlights what is currently known about ATM’s regulation of carbon metabolism, the implication of these pathways in cancer, and the development of ATM inhibitors as therapeutic strategies for cancer.

## Introduction

### Ataxia-Telangiectasia Mutated (ATM)

Ataxia-telangiectasia mutated is a serine/threonine kinase that is recruited to sites of DNA double-strand breaks and signals to various downstream targets to initiate cell cycle arrest and DNA repair (1). Although mainly nuclear, ATM is also found in the cytoplasm and mitochondria (2, 3). In the phosphatidylinositol kinase-related family, ATM consists of many conserved domains and is a tumor suppressor (4). Its kinase domain is flanked by a FAT (FRAT, ATM, and TRRAP) and FATC (C-terminus) domain (5, 6). The function of the FAT domain has yet to be elucidated; however, the FATC domain is essential for kinase activity (7, 8). In addition, ATM has a leucine zipper domain, which is important for its kinase function but not required for dimerization (9). Finally, the N-terminus of ATM encompasses HEAT (*h*untingtin, *e*longation factor 3, *A* subunit of protein phosphatase 2A, and *T*OR1) repeats, which form helices that interact with various macromolecules and play a role in ATM’s kinase function (10, 11).

The activity of ATM in response to DNA damage has been extensively studied as ATM is known as the central regulator of the DNA damage response (DDR). During induction of DNA double-strand breaks, the MRN complex, containing Mre11, Rad50, and Nbs1, binds to the damage site (1). ATM is then activated and autophosphorylates its inactive dimer at serine 1981 (12). Monomeric, active ATM is then recruited to the damage site, where it phosphorylates downstream targets including SMC1, Nbs1, Chk2, BRCA1, and histone H2AX (13, 14). In addition, ATM phosphorylates p53 at serine 15 (15, 16). Activation and repression of ATM’s downstream targets ultimately leads to senescence, genome repair, or apoptosis (17).

*ATM* is the primary gene mutation in ataxia telangiectasia (A-T) (18, 19). A-T is primarily documented as an immunodeficiency and neuronal degeneration disorder affecting 1:40,000–1:100,000 people worldwide (18, 20). Inherited in an autosomal recessive manner, patients typically produce symptoms of delayed development due to neurodegeneration, deficient immune response, and predisposition to cancer. Approximately 10–15% of *ATM* null A-T patients develop childhood leukemia and lymphoma, specifically T-cell prolymphocytic leukemia (21, 22). In addition, patients are predisposed to breast cancer, pancreatic cancer, and melanoma (23). Renwick et al. conducted an unbiased screen in familial breast cancer patients and identified a number of premature truncations and missense variants in *ATM* that predispose patients to cancer (24). Furthermore, immunohistochemical staining of ATM and p53 in pancreatic tumor samples reveal that tumoral loss of ATM with wild-type p53 correlates with a decrease in patient survival, especially in families with a history of pancreatic cancer (25). Finally, somatic *ATM* mutations are implicated in increased melanoma risk (26). Moreover, ATM repairs mitochondrial genome defects, and loss of ATM leads to mitochondrial dysregulation (27). A-T patients have alterations in metabolism, including fluctuations in glucose metabolism (28). In addition, low NAD+ and SIRT1 levels are observed in rat models of A-T (29). These observations lead to the investigation of the role of ATM in metabolism.

### Carbon Metabolism in Cancer

Carbon metabolism is defined as the breakdown of carbon sources, such as glucose and amino acids, to be utilized for cellular energy. Alteration in carbon metabolism is a hallmark of cancer (30). Highly proliferative cancer cells predominantly proceed through aerobic glycolysis rather than the TCA cycle, termed the Warburg effect, requiring high intake of glucose and glutamine (31). This allows cancer cells to compete in a nutrient depleted environment to reduce reactive oxygen species (ROS), generate ATP, and produce dNTPs for proliferation (32, 33). This emphasizes the importance in studying carbon metabolism in cancer and using this knowledge to discover novel, metabolic-based therapeutics.

## Metabolic Roles of ATM

### ATM and ROS

Apart from its role in the DDR, ATM has more recently been implicated in sensing ROS. The role of ATM in ROS sensing has been extensively reviewed (34, 35). Here, we will focus on the coupling of ATM-mediated ROS sensing in cellular metabolism.

In 2011, Cosentino et al. published a pivotal paper linking ROS and the pentose phosphate pathway (PPP) (36). The PPP acts as the *de novo* pathway for deoxyribonucleotide (dNTP) synthesis, important for proliferation and DDR of cancer cells. ATM activates glucose-6-phosphate dehydrogenase (G6PD) through phosphorylation of heat shock protein 27 (Hsp27), which promotes shunting of glycolytic intermediates into the PPP to increase nucleotide synthesis. Furthermore, stimulation of the PPP increases NADPH production, which acts as a cofactor for antioxidants. Together, these data suggest the important role of ATM in the production of dNTPs and NADPH in the proliferation of cancer cells and protection against ROS.

Loss of ATM increases mitochondrial dysregulation, mitochondrial number, and ROS (3). A fraction of ATM localizes to the mitochondria, suggesting that A-T should be further classified as a mitochondrial disorder. Interestingly, this study suggested that the tumor predisposition of A-T patients may be in part due to the mitochondrial dysfunction observed.

Overall, ATM plays a key role in ROS prevention and sensing. The ability of cancer cells to sense ROS through ATM and reprogram metabolism by increasing PPP activity allows for cancer cell survival and resistance to therapy. Cells lacking wild-type ATM are prone to ROS accumulation and oxidative stress. However, the full mechanistic pathway for ATM activation after ROS accumulation is currently unclear.

### ATM and Insulin Signaling

Although beyond the scope of this review, it is important to recognize the evident role of ATM in insulin signaling. The purpose of insulin is to reduce the amount of glucose circulating in the blood and promote cellular uptake of glucose (37). Insulin binds to its respective receptor and recruits GLUT4, a central regulator in glucose homeostasis, to the membrane. GLUT4 transports glucose into the cell where it is used for various processes including glycolysis. A-T patients have an increased risk of developing insulin resistance and type 2 diabetes. Early studies found that A-T patient monocytes have a decreased binding affinity for insulin when compared to unaffected controls (38). Furthermore, ATM signaling through p53 is vital to glucose homeostasis and insulin resistance. Together, these data suggest that ATM regulates glucose homeostasis in part through insulin signaling. Additional information on ATM and insulin signaling can be obtained in several excellent reviews (39–42).

### ATM and Glycolysis

Glycolysis is the main carbon metabolism pathway occurring in the cytosol in which glucose is catabolized into pyruvate through a series of biochemical reactions. Importantly, glycolysis does not require oxygen to proceed and produces a net gain of two ATP molecules and two NADH molecules. Subsequently, in the presence of oxygen, pyruvate enters the mitochondria in the form of acetyl CoA and proceeds through the TCA cycle and oxidative phosphorylation. Conversely, pyruvate is converted to lactic acid in the absence of oxygen or in highly proliferative cancer cells as described above as the Warburg effect (31). ATM phosphorylates and activates the tumor suppressor p53 to regulate cell cycle arrest, apoptosis, senescence, and metabolism (43). p53 suppresses glycolysis through a number of pathways. Interestingly, p53 transcriptionally regulates metabolic genes, including glucose transporters *SLC2A* and *SLC2A4* (encoding for GLUT1 and GLUT4, respectively) (44). p53 also inhibits kinase IKK and targets NFκB, effectively suppressing glycolysis (45). In addition, p53 targets TIGAR, which reduces glycolysis by acting as a fructose-2,6-bisphosphotase (46). It is tempting to speculate that ATM activates p53 to modulate glycolysis through these pathways. Indeed, various DDR proteins are connected to mitochondrial signaling, as discussed in a recent excellent review (47).

### ATM and the PPP

Metabolism is altered in cancer mainly due to the need for nutrients and essential macromolecules in a competing and proliferative environment (32). The PPP is a key pathway in the breakdown of glucose and diverges from glycolysis at glucose-6-phosphate (G6P) (48). Indeed, the increase in proliferation of cancer cells requires the biosynthesis of dNTPs in order to faithfully replicate the genome and repair DNA damage (49, 50). The PPP is essential for *de novo* dNTP synthesis. The PPP produces ribose-5-phosphate, the sugar backbone precursor for purine and pyrimidine synthesis (51). The PPP is divided into the oxidative and non-oxidative pathways. The first irreversible step of the PPP converts NAD+ to NADPH during the conversion of G6P to 6-phosphate-gluconolactone (6PG). The production of NADPH acts as an antioxidant cofactor, protecting the cell from ROS and oxidative stress (52). Together these data suggest an important role of the PPP in the proliferation and reduction of ROS for cancer cell survival.

In response to DNA double-strand breaks, ATM activates Hsp27 and G6PD (36). This interaction increases the flux of G6P to enter the PPP, which increases dNTPs and NADPH to aid DNA repair and reduce ROS, respectively. Conversely, other groups found that ATM negatively regulates the PPP through p53 (52, 53). It is interesting to speculate that there is a balance between positive and negative regulation of the PPP downstream of ATM. It is possible that the amount of DNA damage differentially modulates PPP activity. Under low amounts of DNA damage, Hsp27 is activated to increase dNTP synthesis for DNA repair; however, significant DNA damage accumulation may hyperactivate p53 to inhibit the PPP to fully shut down biosynthetic pathways. Nevertheless, these data support the notion that ATM regulates the PPP to affect dNTP synthesis and NADPH production in cancer cells.

## ATM and Cancer

### Tumor Suppressive Role of ATM in Senescence

Cellular senescence is defined as a stable cell cycle arrest (54) and is, therefore, a potent inhibitor of transformation (55). Senescence also plays a role in aging and is increased in age-related pathologies (56, 57). Senescence occurs due to multiple cellular insults, including telomere shortening, oncogene activation, termed oncogene-induced senescence (OIS), oxidative stress, and DNA damage (54). Senescence is characterized in part by alterations in metabolism (58). Senescence is now considered a reversible process (49, 53, 59–62). Therefore, dissecting how cells escape senescence is critical for understanding the earliest events in tumorigenesis.

One of the underlying mechanisms of OIS is increased replication stress, leading to DNA damage accumulation and cell cycle arrest (63, 64). Replication stress is due to a decrease in dNTP production *via* suppression of ribonucleotide reductase subunit 2 (RRM2), the rate-limiting enzyme in *de novo* dNTP synthesis (49). Replication stress due to decreased dNTPs activates ATM, correlating with senescence induction (53). Loss of ATM rescues senescence through restoration of dNTP levels. This is mediated by a p53-dependent modulation of PPP activity and increased c-myc stability to increase glucose and glutamine consumption. Consistently, a recent study found that pharmacological inhibition of ATM suppresses senescence (65). In this study, pharmacological ATM inhibition also modulated glucose consumption. Together, these data suggest that ATM functions in metabolic regulation and reprogramming in senescent cells.

Oxidative stress induced by ROS can also cause premature senescence in part through DNA damage accumulation. As discussed above, ATM senses and is activated by DNA damage (66). ATM signals through the AKT/p53/p21 pathway to induce senescence in human umbilical vein endothelial cells after oxidative stress (67). In addition, ATM activation is necessary for senescence due to nitric oxide (68). Finally, recent evidence suggests that loss of ATM in A-T mice increases NADPH oxidase 4 (NOX4) expression, leading to increased ROS and senescence (69). Together, these data demonstrate the importance of ATM signaling to induce senescence and suggest that ATM’s role in modulating senescence status offers the possibility of a future therapeutic target in the fields of both aging and cancer.

### ATM Suppresses c-myc

Many cancers upregulate oncogenes that modulate metabolism, including the well-known transcription factor c-myc (70, 71). Specifically, c-myc transcriptionally regulates various enzymes related to metabolic pathways (70, 71). In relation to cancer, c-myc increases the Warburg Effect through upregulation of lactate dehydrogenase, glucose transporters, and pyruvate dehydrogenase kinase. The regulation of c-myc by ATM has just begun to be elucidated. Loss of ATM increases c-myc protein stability, which in turn increases glucose and glutamine consumption (53). Consistently, ATM partially suppresses c-myc-induced lymphomagenesis in mouse models (72, 73). It is interesting to speculate whether this is due to suppression of pro-tumorigenic metabolism. Loss of ATM and c-myc amplification/overexpression are often mutually exclusive in multiple cancer types, suggesting a redundancy in the pathway. Altogether, this suggests an interplay between ATM and c-myc in cancer metabolism.

### ATM Activates AKT

AKT is a well-known serine/threonine kinase that is activated by phosphatidylinositol-3-kinase (PI3K) and regulates many cellular processes related to cancer, including survival, cellular metabolism, and DNA repair (74, 75). ATM activates AKT in response to DNA damage (76–78). Activated AKT then promotes DNA repair (79) and inhibition of AKT decreases DNA repair (80, 81). Consistently, pharmacological inhibition of ATM inhibits AKT phosphorylation and survival in multiple cancer types (82–84). These findings suggest a vital role for AKT in the maintenance of genome integrity, and inhibition of this DNA repair function may result in accumulation of DNA damage and cell death.

AKT also modulates cancer metabolism (85–89). Active AKT increases glucose uptake by recruiting GLUT4 to the plasma membrane (90). In addition, pharmacological inhibition of AKT in primary effusion lymphoma decreases the rate of aerobic glycolysis (91). This suggests that ATM-mediated regulation of AKT activity in cancer reprograms metabolism by increasing glucose uptake and potentially shifting metabolism from aerobic glycolysis to oxidative phosphorylation. It is particularly interesting that ATM-mediated AKT activation may be a double-edged sword, both increasing DNA repair to promote genomic integrity while at the same time increasing pro-tumorigenic metabolism. These data suggest that ATM inhibitors may both alleviate the metabolic changes induced by activated AKT and lead to DNA damage-induced death of cancer cells.

### ATM Regulates p53

p53 is defined as the “guardian of the genome” as it serves to regulate genome stability as a tumor suppressor (92). *TP53* is one of the most mutated genes among all cancers. p53 is a transcription factor that can be activated by ATM (10). Activation of p53 by ATM was originally shown to be important for the regulation of genes essential in apoptosis and DNA repair (93). Further investigation into the interplay between ATM and p53 has revealed its importance in cancer metabolism. p53 regulates many pathways in cellular metabolism, including GLUT recruitment, glycolysis, and oxidative phosphorylation (94). Mutations in p53 lead to metabolic reprogramming in a cancer cells, allowing increased glucose intake through GLUT recruitment to the cell membrane, increased aerobic glycolysis, and decreased oxidative phosphorylation (94, 95). In addition, ATM directly impacts p53-mediated PPP metabolism as discussed above (53). Moreover, ATM loss and p53 mutation are often mutually exclusive in cancer, suggesting that these proteins act in the same pathway to promote cancer cell survival.

### ATM Inhibitors for Cancer Therapy

A variety of ATM inhibitors are currently in pre-clinical and clinical trials for multiple cancer types. ATM inhibitors sensitize various cancer cell lines and tumors *in vitro* and *in vivo* to radiation treatment (83, 96–98). In addition, a phase I clinical trial is currently ongoing with an ATM inhibitor in combination with a PARP inhibitor in advanced cancer patients who are resistant to the standard-of-care (99). Together, these studies have found that cancer cells may be sensitized to DNA damage through inhibition of ATM.

**Figure 1:**
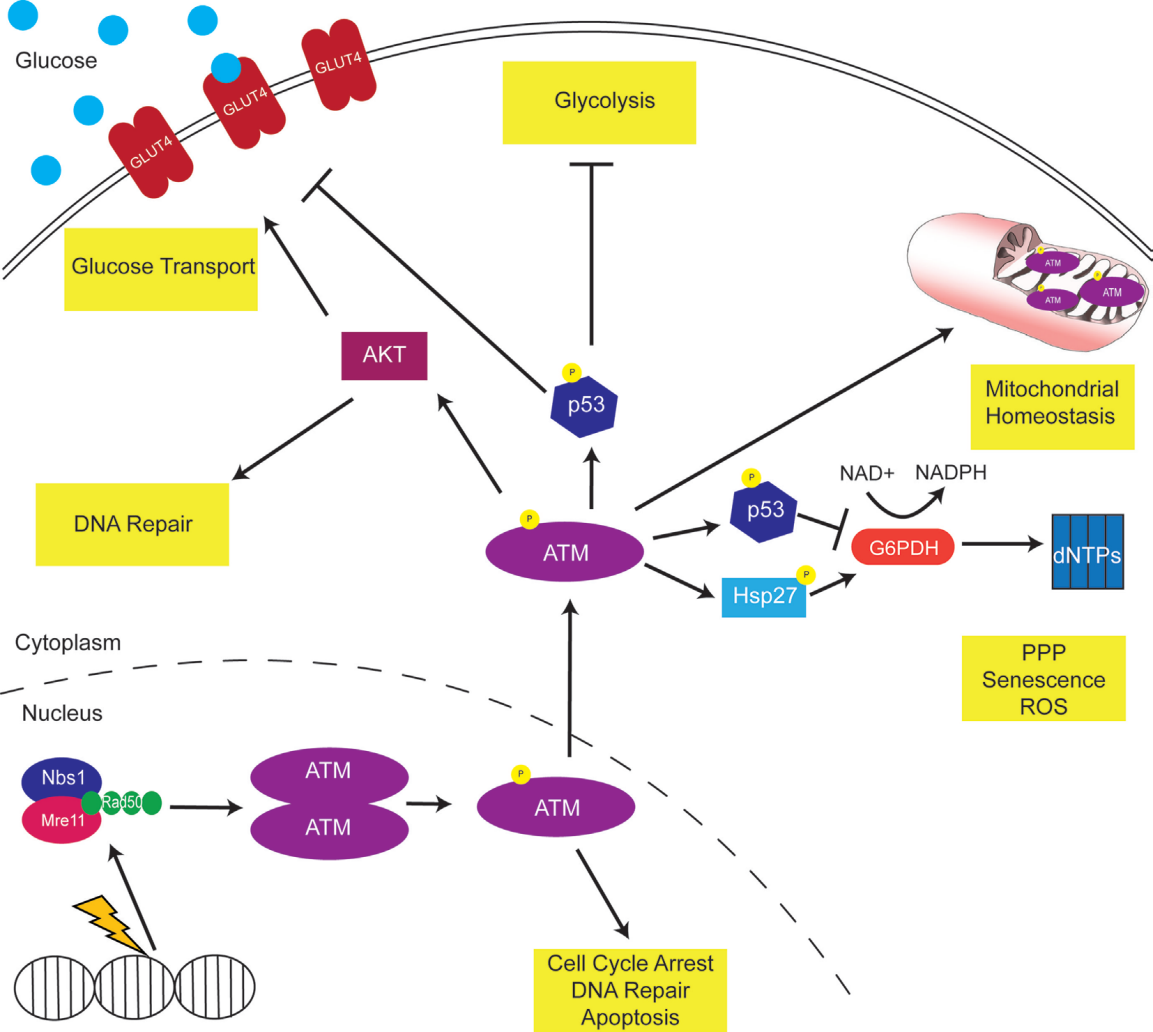
Ataxia-telangiectasia mutated (ATM) modulates cellular metabolism. DNA damage activates ATM to phosphorylate multiple downstream proteins regulate cell cycle arrest, DNA repair, and apoptosis pathways. A non-canonical function of ATM is the regulation of cellular metabolism. Mitochondrial ATM acts to regulate mitochondrial homeostasis by repairing mitochondrial genome defects. ATM activates the tumor suppressor p53, which inhibits GLUT recruitment, glycolysis, and dNTP production. Consistently, p53 targets the oncogene c-myc, inhibiting the TCA cycle and increasing the Warburg effect. In addition, ATM activates AKT to increase GLUT recruitment to the membrane.

As discussed throughout this review, ATM modulates metabolism through various pathways, proteins, and enzymes (Figure 1). Thus, ATM inhibitors may offer a promising way to reprogram the metabolism of cancer cells to make them more vulnerable to anti-metabolic strategies. It will be important to dissect the role of metabolism in pre-clinical and clinical trials using ATM inhibitors.

## Conclusion

Proliferation of cancer cells requires a metabolic shift allowing for an increase in cellular biomass in a highly competitive and nutrient-deprived environment. Although extensively studied for its role in the DDR, non-canonical roles of ATM in metabolic reprogramming have recently been elucidated. ATM modulates carbon metabolism through many pathways that are essential for cancer development, survival, and therapeutic response. Due to their radio- and chemo-sensitizing effects, ATM inhibitors are in pre-clinical and clinical trials as anti-cancer therapeutics. We suggest that ATM inhibitors may also be used to identify metabolic vulnerabilities that could be therapeutically exploited.

## Author Contributions

ED and KA jointly came up with the topic for this mini-review. Both ED and KA wrote and edited the text.

## Conflict of Interest Statement

The authors declare that the research was conducted in the absence of any commercial or financial relationships that could be construed as a potential conflict of interest. The reviewer AT and handling editor declared their shared affiliation.
